# Retraction: TGF-β1 Regulation of Estrogen Production in Mature Rat Leydig Cells

**DOI:** 10.1371/journal.pone.0298690

**Published:** 2024-02-07

**Authors:** Man-Li Liu, Huan Wang, Zong-Ren Wang, Yu-Fen Zhang, Yan-Qiu Chen, Fang-Hong Zhu, Yuan-Qiang Zhang, Jing Ma, Zhen Li

After this article [1] was published, it was noted that in Fig 3A, the lower right region of the TGF β-1 5 ng/ml panel appears similar to the upper left region of the TGF β-1 10 ng/ml panel.

In response, the corresponding author stated that to the best of their knowledge, the results reported in this article were derived from data collected and analyzed following standard scientific protocols at the time of the study. However, they stated that data underlying Fig 3A and a significant portion of other results reported in the article are no longer available.

In light of the unresolved issues about Fig 3, the unavailability of data, and to uphold standards of scientific rigor, the corresponding author requested to retract the article. Although the authors stand by the article’s findings, they are no longer able to be accountable for the reported work as is required by the PLOS Authorship policy.

In light of the above issues, the authors retract this article.

All authors agree with retraction and apologize for the issues with the published article.
